# Study of Cyberbullying among Adolescents in Recent Years: A Bibliometric Analysis

**DOI:** 10.3390/ijerph18063016

**Published:** 2021-03-15

**Authors:** Ana Belén Barragán Martín, María del Mar Molero Jurado, María del Carmen Pérez-Fuentes, María del Mar Simón Márquez, África Martos Martínez, Maria Sisto, José Jesús Gázquez Linares

**Affiliations:** 1 Department of Psychology, Faculty of Psychology, University of Almería, 04120 Almería, Spain; abm410@ual.es (A.B.B.M.); mpf421@ual.es (M.d.C.P.-F.); msm112@ual.es (M.d.M.S.M.); amm521@ual.es (Á.M.M.); ms168@ual.es (M.S.); jlinares@ual.es (J.J.G.L.); 2 Department of Psychology, Universidad Autónoma de Chile, Providencia 7500000, Chile

**Keywords:** cyberbullying, adolescents, bibliometric analysis, co-authorship network, publications

## Abstract

In recent years, cyberbullying has been recognized as a severe public health problem and is drawing growing interest. The objective of this study was to perform a bibliometric analysis of the scientific production on adolescent cyberbullying in the last decade. A search for publications was made in the Web of Science database, where the 1530 documents identified were analyzed with BibExcel software and visualized using the Pajek and VOSviewer tools. The predominant language in the publications was English, followed by Spanish. The publication rate was shown to have increased in recent years. The journal “*Computers in Human Behavior*” had the highest production. The repercussion of new technologies on this phenomenon has been felt, and research groups have enlarged their production in response to the problem. A systematic review and/or meta-analysis examining the contents of the studies identified and the variables related to this problem is therefore necessary. This could identify a point of reference for research in this field and a basis for future reviews of its development and progress over time.

## 1. Introduction

Social relations during adolescence are essential to individual psychological well-being [1] and social development [2], because it is when one begins to learn to interact with society, to create the first friendships, and to resolve the first conflicts, which can sometimes turn violent [3,4], as in bullying [5,6]. Such situations usually have negative repercussions on the lives of young people who have been involved in them. However, bullying takes place not only in the school. With the development of new information and communication technologies, cyberbullying has emerged [7]. The strong impact of the constant increment and dependence on ICTs has changed the content, habits, and forms of interpersonal relations [8]. Both bullying and cyberbullying have been recognized as a severe public health problem [9,10], as they represent a threat to the development of the mental health and well-being of children and adolescents [11]. Both refer to a type of violence, in which cyberbullying specifically uses ICTs to harass peers [12,13]. Cyberbullying victims are usually more emotionally and more severely affected than victims of traditional bullying. In most cases, the two phenomena coincide, but cyberbullying is not often discussed separately [14]. More so, there is co-occurrence between bullying and cyberbullying, in which some adolescents are cyberaggression victims [15].

Some studies suggest a prevalence of cyberbullying of 6.5% to 35.4% in Europe and the United States [16], although the prevalence of cyberbullying among adolescents varies depending on the factors evaluated [17,18]. Its appearance is linked to such traits as low self-esteem [19]; low affective and cognitive empathy [20]; social and psychological maladjustments [21,22]; states of depression, anxiety, and anger [23]; low academic performance [24]; school absenteeism [25]; and others. 

There is no exact delimitation of the main risk and protection factors that predict cyberbullying; however, some individual, family, and social variables can be found [26]. Lower empathy and male sex [27] are mentioned as predictor variables, and physical aptitude as a possible protective factor [28]. In the family, indulgent and authoritarian parenting styles influence aggressive adolescent behavior, while styles showing the best results involve the warmth and affect of parents toward their children [29]. Even though there is a close relationship with social influences during adolescence, parenting styles are more predominant in the development of aggressiveness [30]. However, social influence can also have repercussions on the intention and behavior of cyberbullying [31], along with the use of social networks and texting, which are predictors of occasional cyberbullying [26,32].

The scientific production on this phenomenon has been reviewed and analyzed in systematic reviews of databases [16,33] by bibliometric analysis. Bibliometric analysis can identify elements in the literature, such as the most productive authors, countries, institutions, and journals within an area of study, as well as trends in production and collaboration networks [34], and bibliometric mapping provides a description of publications on a structural level by visualizing production [35]. Herrera-López, Romera, and Ortega-Ruiz [36] performed a bibliometric study in Latin America, identifying bibliographic production on bullying and cyberbullying, and emphasized more research on the first phenomenon than on the second. Other studies that have reviewed cyberbullying in Spanish journals [37] have shown social networks to be the mobile communication tools with the most immediacy among youths, and have underlined the important role of intervention to prevent and reduce this type of violence [38,39]. English, the main language of communication and publication in research [40], is the predominant language of these studies. 

In view of the above, the objective of this study was to analyze the evolution of this topic by employing the bibliometric method to acquire an overview of cyberbullying in the last 10 years through indicators, such as the number of publications, types of documents, countries and institutions, authors, and so forth. We also wanted to identity the trends in publications on cyberbullying in adolescents during this period.

## 2. Materials and Methods

Bibliometric analyses have become an additional tool for determining the quality of scientific literature and its repercussion on the population [41].

### 2.1. Selecting the Database

First, Web of Science, Scopus, and Psychology Database were compared for their use and relevance in the field, employing the terms “cyberbullying,” “adolescent,” “teenagers,” and “adolescence.” The search formula was (cyberbullying AND (adolescent OR youth OR teenagers OR adolescence)). The search for publications was limited to the period from 2010 to 2020.

After this preliminary analysis in each of the databases, we found that there were a similar number of documents in Scopus (1046) and Psychology Database (1017). However, there was a significant difference in scientific production related to the subject in Web of Science, where results were much higher.

### 2.2. Data Collection and Search Strategy

The Web of Science (WOS) database was therefore used in this study, limiting the search to publications from 1 January 2010 to 1 January 2020. The search equation was TS = (cyberbullying AND (adolescent OR youth OR teenagers OR adolescence)), applying filters, such as type of document, and selecting articles in journals, whether open access or not. When all the filters had been selected, the results were imported from the database as unformatted text for later analysis. A total of 1530 documents were found.

### 2.3. Inclusion and Exclusion Criteria

The inclusion criteria were (a) study on cyberbullying; (b) study population of adolescents, young people, or youth; (c) publication from 1 January 2010 to 1 January 2020; and (d) language being English or Spanish.

The exclusion criteria were studies that (a) were not on adolescence or about young university students and (b) did not use English or Spanish as publication language.

After applying the filters and inclusion and exclusion criteria, the 1530 documents the search had produced were reduced to 1276.

### 2.4. Data Analysis

The files were downloaded from the Web of Science database separately as plain text, then combined and transformed so they could be read by BibExcel [42]. This program can manipulate and preprocess a large volume of data at the same time. Microsoft Excel (Microsoft Corporation, Redmond, WA, USA programs) was also used to count the data on language, journals, and countries and to calculate the trend of publications in cyberbullying in adolescents. All the analyses were performed on the 1276 documents, except for the language analysis, which was done on all 1530 articles. Pajek (developed by Andrej Mrvar and Vladimir Batagelj, University of Ljubljana, Slovenia) was used for the construction and visualization of the networks [43], and VOSviewer [44] (The Centre for Science and Technology Studies, Leiden, Netherlands) constructed the co-authorship and word networks.

## 3. Results

A total of 1530 files related to cyberbullying in adolescents found in the Web of Science database were analyzed with the following indicators to find out the type of studies done and the trend in the subject:

The predominant language of the publications, with 92.71% (*n* = 1183), was English, followed by Spanish with 8% (*n* = 102), French with 0.55 (*n* = 7), Portuguese with 0.31% (*n* = 4), and others with 0.31% (*n* = 4).

**Figure 1:**
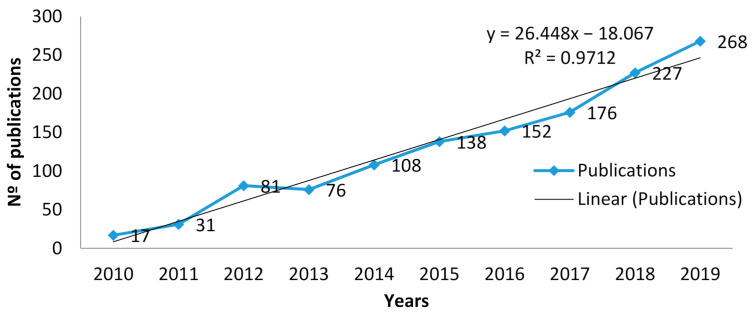
Scientific production on adolescent cyberbullying and its trend from 2010 to 2020.

Figure 1 presents the international scientific production of articles on adolescent cyberbullying, showing an increase in publications in the last 10 years. The first 4 years from 2010 through 2013 were less productive than the rest of the years, although there was an increase of 50 articles in 2011/2012. Starting in 2014, production began to increase, and the most productive period was 2017–2018, in which 51 more studies were published than the year before. In the following years, the trend in scientific production continued to rise (R^2^ = 0.971).

**Figure 2:**
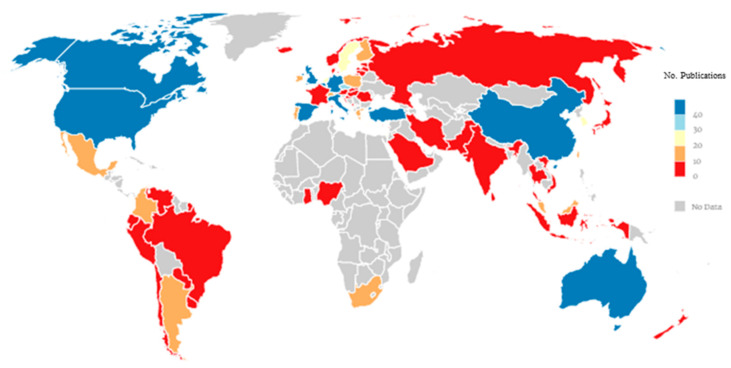
Trends in publications on adolescent cyberbullying by country.

Figure 2 shows the distribution of documents by country for the period from 2010 through 2019. The country with the most publications was the United States (355), followed by Spain (214), England (100), Australia (75), Canada (72), China (61), and Italy (60). Spain was the country with the second highest production on adolescent cyberbullying.

**Figure 3:**
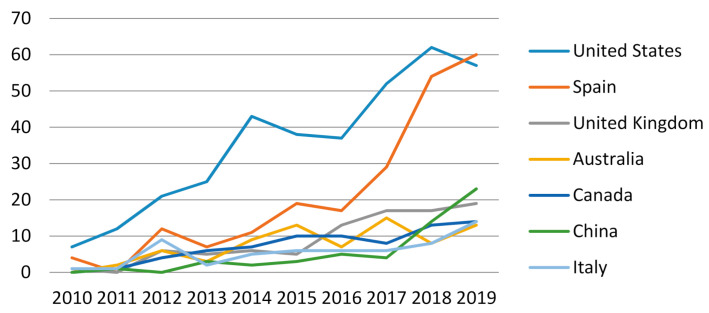
Trend in publications in countries with the highest production rates.

Figure 3 shows the trend in publications on adolescent cyberbullying in each of the seven countries with the highest percentages of scientific production. The United States and Spain were the countries with the greatest increases.

The general category with the most publications was social sciences, followed by scientific technology, life science and biomedicine, technology, and areas such as physical sciences, art, and humanities.

A total of 501 journals published articles on adolescent cyberbullying. Table 1 shows the 18 journals with the most articles. *Computers in Human Behavior* (101) published the most, followed by *Cyberpsychology, Behavior, and Social Networking* (43) and the *International Journal of Environmental Research and Public Health* (42). These journals rank in the first and second quartile in the SJR (SCImago Journal & Country Rank), which is an indicator evaluating the importance and quality of a journal within its area. Most of the documents were published in journals in the United States, the United Kingdom, Spain and the Netherlands.

**Figure 4:**
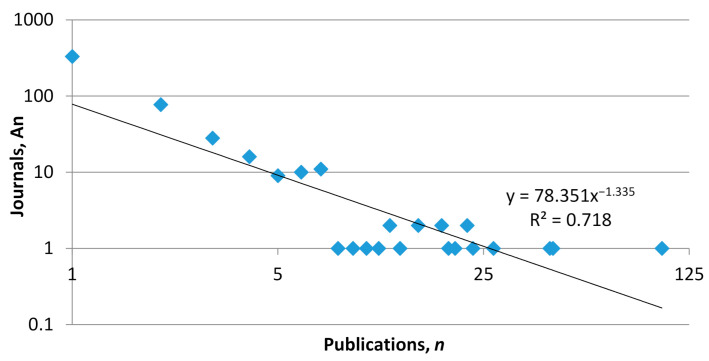
Plot of Lotka’s law applied to journal productivity.

Journal productivity on adolescent cyberbullying was compared by applying Lotka’s law, which showed that just 1 journal had 101 documents related to the subject, while 330 journals had only published 1 article during this period. The curve found was similar to an R^2^ near 1, 0.718 (Figure 4).

**Figure 5:**
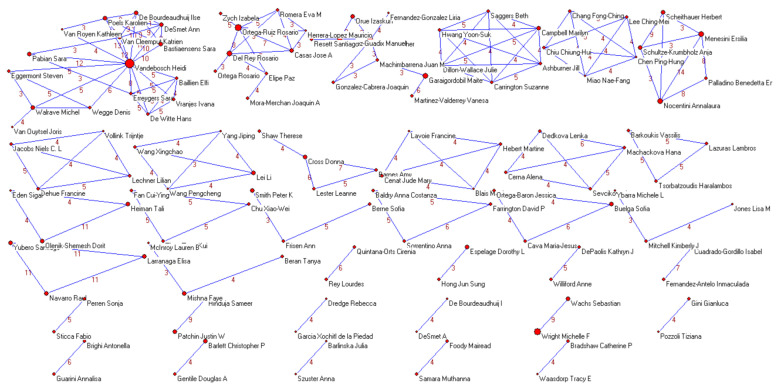
Co-authorship network. Map of collaboration by co-authors.

Figure 5 shows the co-authorship networks only of those authors who collaborated in at least four publications on adolescent cyberbullying, as the number of co-authorships was reduced for visualization with Pajek. The connection between authors is shown by the line that connects them, whether they are authors or co-authors of the same study. The transitivity coefficient of the co-authorship network was 0.82.

**Figure 6:**
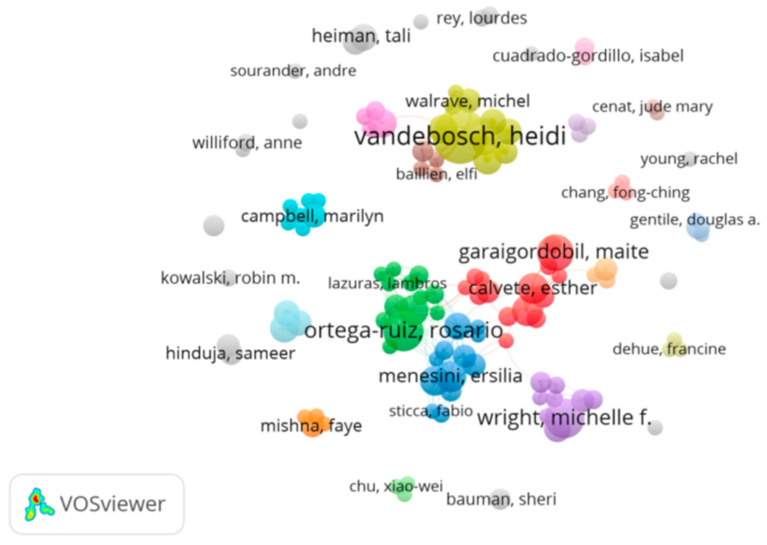
Authorship plot by number of publications.

Figure 6 also shows the authors with the most publications on adolescent cyberbullying and their interaction. In this figure, the authors are the colored nodes or points, where the size of the node represents the number of articles published from 2010 through 2020. The author correlationships show how they group together for publishing. Clusters of the same color show the publication collaboration network, which includes a total of 121 authors distributed in 32 clusters, where the yellow cluster, made up of 10 authors led by Heidi Vandebosch, is the largest. This is followed by the red cluster comprising 15 authors, where Maite Garaigordobil and Esther Calvete are the authors with the most publications. In the green cluster, consisting of 12 authors, Rosario Ortega-Ruiz is outstanding with 28 articles. Ersilia Menesini, in the blue cluster of 12 authors, is also outstanding. The rest of the clusters are made up of fewer than 10 authors. The author with the most publications and the strongest link is Heidi Vandebosch, with a total of 37. Sameer Hinduja and Justin W. Patchin were cited the most with a total of 1158 citations.

Table 2 shows a selection of the main authors, where the total strength of the co-authorship bonds with other authors may be observed. Heidi Vandebosch is the strongest link, in addition to being the author with the most publications on the subject. She is followed by Rosario Ortega, who not only has 28 articles on cyberbullying to her name, but also was cited 803 times. Likewise, the repercussion of authors such as Sameer Hinduja and Justin W. Patchin, who, with only 9 publications, were cited 1158 times, is impressive. Peter K. Smith is also outstanding with 930 citations.

**Figure 7:**
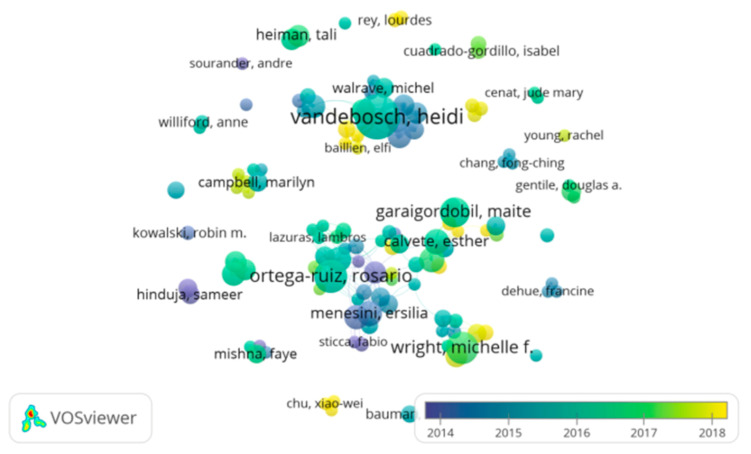
Visualization of authors by year.

Figure 7 shows the year articles were published by color, where the predominant period is 2016–2017, followed by 2015–2016.

**Figure 8:**
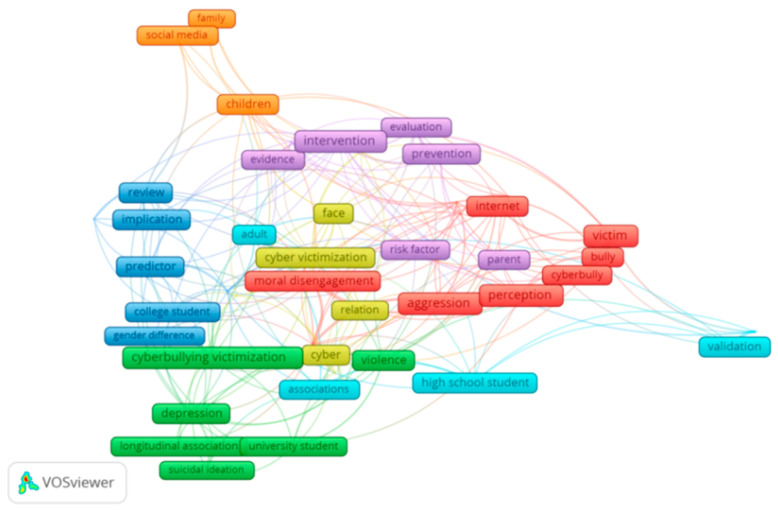
Coincidence of words in titles of publications on adolescent cyberbullying.

Figure 8 shows the concurrence of main words in the titles of the publications analyzed. The cluster colors show the coincidence of terms in the titles. That is, it clusters the words that appear together the most in document titles: cyberbully, cyber, cyber victimization, cyberbullying victimization, children, college student, risk factor, predictor.

## 4. Discussion

The objective of this study was to find out the current panorama of international scientific bibliographic production related to adolescent cyberbullying in a bibliometric approach. This phenomenon has been recognized as a public health problem [9,10]. Its repercussions on society have led to an increase in the number of scientific publications, with a prevalence that varies from 6.5% to 35.4% in Europe and the United States [16]. In the last 10 years, there has been a continuous upward trend in the number of publications, and the most productive period was from 2017 to 2018. Furthermore, scientific production increased rapidly from 2011, where the growth in number of publications coincided with the boom of social networking and cell phones and the appearance of a new type of multitasking consumer [39]. In spite of these data, investigative production on this phenomenon is much lower than on bullying [36], as some authors do not consider it a different phenomenon, but a type of bullying [14]. 

The predominant language was English, as already described in other studies, as it is the main language of publication and communication in research [40], followed by Spanish. In line with other reviews and analyses of this phenomenon, the United States was the country with the highest scientific production, followed by Spain, both standing out from the rest as the main drivers of publications on adolescent cyberbullying [33,37]. The category with the highest production related to the subject was social sciences, being the area that studies social behavior.

Several main journals were identified, as in any field of research, not only due to the frequency of publications, but also due to the mentions they received, which are equally important. *Computers in Human Behavior,* which is an academic journal on the use of computers in psychology and the psychological impact of their use in society, is the journal with the largest number of publications on the subject of study, standing out from the rest by a wide difference, because of its production statistics, given the large number of volumes it publishes yearly.

The analysis of authors demonstrated that many researchers are approaching the subject of adolescent cyberbullying. Thirty-two groups were identified, and in most groups, the collaboration network extends to researchers in other countries. These groups are made up of researchers from areas where the cyberbullying prevalence rates are highest, such as Europe and the United States [16], and therefore, their interest in the subject as the focus of research is higher.

This study has some limitations, one of which is the restriction of the search to a single database, Web of Science, and the choice of the descriptors used for the search. Although it was attempted to integrate all the terms related to adolescence, some studies may have been excluded because they were otherwise described. Therefore, future studies should widen both search terms and databases. In addition, as a potential line of future research, we recommend the need to carry out a systematic review and/or meta-analysis, analyzing the contents of the studies carried out, and the variables related to this problem. A reference point for research in this field, and as a basis for future reviews of its development and progress over time, could thus be found. 

## 5. Conclusions

In spite of the limitations, these results have a significant implication for understanding how new technologies and social networking tools, when poorly managed and combined with psychological and social maladjustments, can lead to negative repercussions on the lives of individuals. This bibliometric analysis also enabled us to communicate the key findings to the researchers, who can start up the prevention and intervention mechanisms necessary.

Moreover, the reason that the production related to adolescent cyberbullying is not decreasing, but on the contrary is growing, is that institutions and researchers are finding that it has become a severe and growing public health problem.

Therefore, the results of this study have practical implications in education and health, especially in identifying areas for future research that should be considered for the study and design of interventions. In addition, information on the areas where most studies are concentrated will enable the identification of less studied areas, where it is possible to continue exploring other resources for education.

## Figures and Tables

**Table 1:** Selection of journals with the most publications on adolescent cyberbullying.

Journal	No. of Publications	Quartile	SJR (2018)	H Index	Country
*Computers in Human Behavior*	101	Q1	1.71	137	United Kingdom
*Cyberpsychology, Behavior, and Social Networking*	43	Q1	1.31	119	United States
*International Journal of Environmental Research and Public Health*	42	Q2	0.82	78	Switzerland
*Journal of Interpersonal Violence*	27	Q1	1.17	93	United States
*Aggressive Behavior*	23	Q1	1.47	82	United States
*Frontiers in Psychology*	22	Q1	1	81	Switzerland
*Journal of Adolescence*	22	Q1	1.09	101	United States
*Children and Youth Services Review*	20	Q1	0.75	77	United Kingdom
*Journal of School Violence*	19	Q1	1.09	32	United States
*Journal of Adolescent Health*	18	Q1	2.35	142	Netherlands
*Journal of Youth and Adolescence*	18	Q1	1.52	101	Netherlands
*Psicothema*	15	Q2	0.64	47	Spain
*BMC Public Health*	15	Q1	1.38	117	United Kingdom
*Violence and Victims*	13	Q1	0.57	76	United States
*Journal of Youth Studies*	12	Q1	1	46	United States
*School Psychology International*	12	Q2	0.62	49	United States
*Comunicar*	11	Q1	0.85	26	Spain
*Journal of School Health*	10	Q1	0.86	75	United States

**Table 2:** Number of documents and citations and strength of co-authorship links.

Author	Documents	Citations	Total Bond Strength
Vandebosch, Heidi	37	518	107
Ortega-Ruiz, Rosario	28	803	57
Wright, Michelle F.	22	197	15
Garaigordobil, Maite	19	253	10
Menesini, Ersilia	15	570	35
Calvete, Esther	15	589	22
Gamez-Guadix, Manuel	15	372	15
Nocentini, Annalaura	14	513	35
Del Rey, Rosario	14	331	31
Poels, Karolien	13	300	53
Pabian, Sara	13	210	29
Navarro, Raul	13	199	22
Smith, Peter K.	12	930	20
Van Cleemput, Katrien	12	331	53
Heiman, Tali	12	159	11
Wachs, Sebastian	12	70	11
De Bourdeaudhuij, Ilse	11	290	50
Desmet, Ann	11	290	50
Olenik-Shemesh, Dorit	11	159	11
Larranaga, Elisa	11	142	22
Yubero, Santiago	11	142	22
Bastiaensens, Sara	10	276	50
Walrave, Michel	10	253	13
Casas, Jose A.	10	228	27
Barlett, Christopher P.	10	100	5
Campbell, Marilyn	10	88	17
Hinduja, Sameer	9	1158	9
Patchin, Justin W.	9	1158	9
Orue, Izaskun	9	517	14
Scheithauer, Herbert	9	344	23
Schultze-Krumbholz, Anja	9	344	23
Buelga, Sofia	9	197	10
Espelage, Dorothy I.	9	174	6
Lei, Li	9	56	12
Mishna, Faye	9	54	8

## Data Availability

The data that support the findings of this study are available from the corresponding author upon reasonable request.
